# Speech perception as an active cognitive process

**DOI:** 10.3389/fnsys.2014.00035

**Published:** 2014-03-17

**Authors:** Shannon L. M. Heald, Howard C. Nusbaum

**Affiliations:** Department of Psychology, The University of ChicagoChicago, IL, USA

**Keywords:** speech, perception, attention, learning, active processing, theories of speech perception, passive processing

## Abstract

One view of speech perception is that acoustic signals are transformed into representations for pattern matching to determine linguistic structure. This process can be taken as a statistical pattern-matching problem, assuming realtively stable linguistic categories are characterized by neural representations related to auditory properties of speech that can be compared to speech input. This kind of pattern matching can be termed a passive process which implies rigidity of processing with few demands on cognitive processing. An alternative view is that speech recognition, even in early stages, is an active process in which speech analysis is attentionally guided. Note that this does not mean consciously guided but that information-contingent changes in early auditory encoding can occur as a function of context and experience. Active processing assumes that attention, plasticity, and listening goals are important in considering how listeners cope with adverse circumstances that impair hearing by masking noise in the environment or hearing loss. Although theories of speech perception have begun to incorporate some active processing, they seldom treat early speech encoding as plastic and attentionally guided. Recent research has suggested that speech perception is the product of both feedforward and feedback interactions between a number of brain regions that include descending projections perhaps as far downstream as the cochlea. It is important to understand how the ambiguity of the speech signal and constraints of context dynamically determine cognitive resources recruited during perception including focused attention, learning, and working memory. Theories of speech perception need to go beyond the current corticocentric approach in order to account for the intrinsic dynamics of the auditory encoding of speech. In doing so, this may provide new insights into ways in which hearing disorders and loss may be treated either through augementation or therapy.

In order to achieve flexibility and generativity, spoken language understanding depends on active cognitive processing (Nusbaum and Schwab, 1986; Nusbaum and Magnuson, 1997). Active cognitive processing is contrasted with passive processing in terms of the control processes that organize the nature and sequence of cognitive operations (Nusbaum and Schwab, 1986). A passive process is one in which inputs map directly to outputs with no hypothesis testing or information-contingent operations. Automatized cognitive systems (Shiffrin and Schneider, 1977) behave as though passive, in that stimuli are mandatorily mapped onto responses without demand on cognitive resources. However it is important to note that cognitive automatization does not have strong implications for the nature of the mediating control system such that various different mechanisms have been proposed to account for automatic processing (e.g., Logan, 1988). By comparison, active cognitive systems however have a control structure that permits “information contingent processing” or the ability to change the sequence or nature of processing in the context of new information or uncertainty. In principle, active systems can generate hypotheses to be tested as new information arrives or is derived (Nusbaum and Schwab, 1986) and thus provide substantial cognitive flexibility to respond to novel situations and demands.

## Active and passive processes

**Figure 1:**
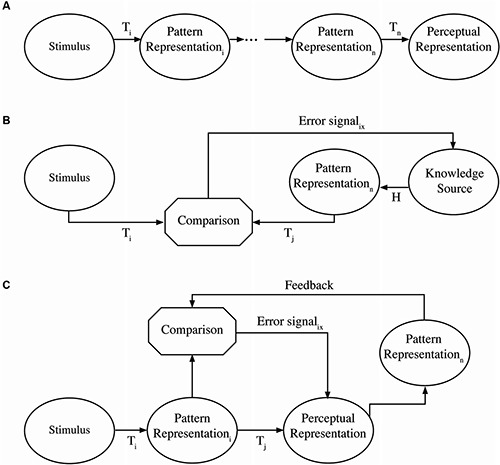
**Schematic representation of passive and active processes**. The top panel **(A)** represents a passive process. A stimulus presented to sensory receptors is transformed through a series of processes (Ti) into a sequence of pattern representations until a final perceptual representation is the result. This could be thought of as a pattern of hair cell stimulation being transformed up to a phonological representation in cortex. The middle panel **(B)** represents a top-down active process. Sensory stimulation is compared as a pattern to hypothesized patterns derived from some knowledge source either derived from context or expectations. Error signals from the comparison interact with the hypothesized patterns until constrained to a single interpretation. The generation of hypothesized patterns may be in parallel or accomplished sequentially. The bottom panel **(C)** represents a bottom-up active process in which sensory stimulation is transformed into an initial pattern, which can be transformed into some representation. If this representation is sensitive to the unfolding of context or immediate perceptual experience, it could generate a pattern from the immediate input and context that is different than the initial pattern. Feedback from the context-based pattern in comparison with the initial pattern can generate an error signal to the representation changing how context is integrated to produce a new pattern for comparison purposes.

The distinction between active and passive processes comes from control theory and reflects the degree to which a sequence of operations, in this case neural population responses, is contingent on processing outcomes (see Nusbaum and Schwab, 1986). A passive process is an open loop sequence of transformations that are fixed, such that there is an invariant mapping from input to output (MacKay, 1951, 1956). Figure 1A illustrates a passive process in which a pattern of inputs (e.g., basilar membrane responses) is transmitted directly over the eighth nerve to the next population of neurons (e.g., in the auditory brainstem) and upward to cortex. This is the fundamental assumption of a number of theories of auditory processing in which a fixed cascade of neural population responses are transmitted from one part of the brain to the other (e.g., Barlow, 1961). This type of system operates the way reflexes are assumed to operate in which neural responses are transmitted and presumably transformed but in a fixed and immutable way (outside the context of longer term reshaping of responses). Considered in this way, such passive processing networks should process in a time frame that is simply the sum of the neural response times, and should not be influenced by processing outside this network, functioning something like a module (Fodor, 1983). In this respect then, such passive networks should operate “automatically” and not place any demands on cognitive resources. Some purely auditory theories seem to have this kind of organization (e.g., Fant, 1962; Diehl et al., 2004) and some more classical neural models (e.g., Broca, 1865; Wernicke, 1874/1977; Lichtheim, 1885; Geschwind, 1970) appear to be organized this way. In these cases, auditory processes project to perceptual interpretations with no clearly specified role for feedback to modify or guide processing.

By contrast, active processes are variable in nature, as network processing is adjusted by an error-correcting mechanism or feedback loop. As such, outcomes may differ in different contexts. These feedback loops provide information to correct or modify processing in real time, rather than retrospectively. Nusbaum and Schwab (1986) describe two different ways an active, feedback-based system may be achieved. In one form, as illustrated in Figure 1B, expectations (derived from context) provide a hypothesis about a stimulus pattern that is being processed. In this case, sensory patterns (e.g., basilar membrane responses) are transmitted in much the same way as in a passive process (e.g., to the auditory brainstem). However, descending projections may modify the nature of neural population responses in various ways as a consequence of neural responses in cortical systems. For example, top-down effects of knowledge or expectations have been shown to alter low level processing in the auditory brainstem (e.g., Galbraith and Arroyo, 1993) or in the cochlea (e.g., Giard et al., 1994). Active systems may occur in another form, as illustrated in Figure 1C. In this case, there may be a strong bottom-up processing path as in a passive system, but feedback signals from higher cortical levels can change processing in real time at lower levels (e.g., brainstem). An example of this would be the kind of observation made by Spinelli and Pribram (1966) in showing that electrical stimulation of the inferotemporal cortex changed the receptive field structure for lateral geniculate neurons or Moran and Desimone’s (1985) demonstration that spatial attentional cueing changes effective receptive fields in striate and extrastriate cortex. In either case, active processing places demands on the system’s limited cognitive resources in order to achieve cognitive and perceptual flexibility. In this sense, active and passive processes differ in the cognitive and perceptual demands they place on the system.

Although the distinction between active and passive processes seems sufficiently simple, examination of computational models of spoken word recognition makes the distinctions less clear. For a very simple example of this potential issue consider the original Cohort theory (Marslen-Wilson and Welsh, 1978). Activation of a set of lexical candidates was presumed to occur automatically from the initial sounds in a word. This can be designated as a passive process since there is a direct invariant mapping from initial sounds to activation of a lexical candidate set, i.e., a cohort of words. Each subsequent sound in the input then deactivates members of this candidate set giving the appearance of a recurrent hypothesis testing mechanism in which the sequence of input sounds deactivates cohort members. One might consider this an active system overall with a passive first stage since the initial cohort set constitutes a set of lexical hypotheses that are tested by the use of context. However, it is important to note that the original Cohort theory did not include any active processing at the phonemic level, as hypothesis testing is carried out in the context of word recognition. Similarly, the architecture of the Distributed Cohort Model (Gaskell and Marslen-Wilson, 1997) asserts that activation of phonetic features is accomplished by a passive system whereas context interacts (through a hidden layer) with the mapping of phonetic features onto higher order linguistic units (phonemes and words) representing an interaction of context with passively derived phonetic features. In neither case is the activation of the features or sound input to linguistic categorization treated as hypothesis testing in the context of other sounds or linguistic information. Thus, while the Cohort models can be thought of as an active system for the recognition of words (and sometimes phonemes), they treat phonetic features as passively derived and not influenced from context or expectations.

This is often the case in a number of word recognition models. The Shortlist models (Shortlist: Norris, 1994; Shortlist B: Norris and McQueen, 2008) assume that phoneme perception is a largely passive process (at least it can be inferred as such by lack of any specification in the alternative). While Shortlist B uses phoneme confusion data (probability functions as input) and could in principle adjust the confusion data based on experience (through hypothesis testing and feedback), the nature of the derivation of the phoneme confusions is not specified; in essence assuming the problem of phoneme perception is solved. This appears to be common to models (e.g., NAM, Luce and Pisoni, 1998) in which the primary goal is to account for word perception rather than phoneme perception. Similarly, the second Trace model (McClelland and Elman, 1986) assumed phoneme perception was passively achieved albeit with competition (not feedback to the input level). It is interesting that the first Trace model (Elman and McClelland, 1986) did allow for feedback from phonemes to adjust activation patterns from acoustic-phonetic input, thus providing an active mechanism. However, this was not carried over into the revised version. This model was developed to account for some aspects of phoneme perception unaccounted for in the second model. It is interesting to note that the Hebb-Trace model (Mirman et al., 2006a), while seeking to account for aspects of lexical influence on phoneme perception and speaker generalization did not incorporate active processing of the input patterns. As such, just the classification of those inputs was actively governed.

This can be understood in the context schema diagrammed in Figure 1. Any process that maps inputs onto representations in an invariant manner or that would be classified as a finite-state deterministic system can be considered passive. A process that changes the classification of inputs contingent on context or goals or hypotheses can be considered an active system. Although word recognition models may treat the recognition of words or even phonemes as an active process, this active processing is not typically extended down to lower levels of auditory processing. These systems tend to operate as though there is a fixed set of input features (e.g., phonetic features) and the classification of such features takes place in a passive, automatized fashion.

By contrast, Elman and McClelland (1986) did describe a version of Trace in which patterns of phoneme activation actively changes processing at the feature input level. Similarly, McClelland et al. (2006) described a version of their model in which lexical information can modify input patterns at the subphonemic level. Both of these models represent active systems for speech processing at the sublexical level. However, it is important to point out that such theoretical propositions remain controversial. McQueen et al. (2006) have argued that there are no data to argue for lexical influences over sublexical processing, although Mirman et al. (2006b) have countered this with empirical arguments. However, the question of whether there are top-down effects on speech perception is not the same as asking if there are active processes governing speech perception. Top-down effects assume higher level knowledge constrains interpretations, but as indicated in Figure 1C, there can be bottom-up active processing where by antecedent auditory context constrains subsequent perception. This could be carried out in a number of ways. As an example, Ladefoged and Broadbent (1957) demonstrated that hearing a context sentence produced by one vocal tract could shift the perception of subsequent isolated vowels such that they would be consistent with the vowel space of the putative speaker. Some have accounted for this result by asserting there is an automatic auditory tuning process that shifts perception of the subsequent vowels (Huang and Holt, 2012; Laing et al., 2012). While the behavioral data could possibly be accounted for by such a simple passive mechanism, it might also be the case the auditory pattern input produces constraints on the possible vowel space or auditory mappings that might be expected. In this sense, the question of whether early auditory processing of speech is an active or passive process is still a point of open investigation and discussion.

It is important to make three additional points in order to clarify the distinction between active and passive processes. First, a Bayesian mechanism is not on its own merits necessarily active or passive. Bayes rule describes the way different statistics can be used to estimate the probability of a diagnosis or classification of an event or input. But this is essentially a computation theoretic description much in the same way Fourier’s theorem is independent of any implementation of the theorem to actually decompose a signal into its spectrum (cf. Marr, 1982). The calculation and derivation of relevant statistics for a Bayesian inference can be carried out passively or actively. Second, the presence of learning within a system does not on its own merits confer active processing status on a system. Learning can occur by a number of algorithms (e.g., Hebbian learning) that can be implemented passively. However to the extent that a system’s inputs are plastic during processing, would suggest whether an active system is at work. Finally, it is important to point out that active processing describes the architecture of a system (the ability to modify processing on the fly based on the processing itself) but not the behavior at any particular point in time. Given a fixed context and inputs, any active system can and likely would mimic passive behavior. The detection of an active process therefore depends on testing behavior under contextual variability or resource limitations to observe changes in processing as a consequence of variation in the hypothesized alternatives for interpretation (e.g., slower responses, higher error rate or confusions, increase in working memory load).

## Computational need for active control systems in speech perception

Understanding how and why active cognitive processes are involved in speech perception is fundamental to the development of a theory of speech perception. Moreover, the nature of the theoretical problems that challenge most explanations of speech perception are structurally similar to some of the theoretical issues in language comprehension when considered more broadly. In addition to addressing the basis for language comprehension broadly, to the extent that such mechanisms play a critical role in spoken language processing, understanding their operation may be important to understanding both the effect of hearing loss on speech perception as well as suggesting ways of remediating hearing loss. If one takes an overly simplified view of hearing (and thus damage to hearing resulting in loss) as an acoustic-to-neural signal transduction mechanism comparable to a microphone-amplifier system, the simplifying assumptions may be very misleading. The notion of the peripheral auditory system as a passive acoustic transducer leads to theories that postulate passive conversion of acoustic energy to neural signals and this may underestimate both the complexity and potential of the human auditory system for processing speech. At the very least, early auditory encoding in the brain (reflected by the auditory brainstem response) is conditioned by experience (Skoe and Kraus, 2012) and so the distribution of auditory experiences shapes the basic neural patterns extracted from acoustic signals. However, it is appears that this auditory encoding is shaped from the top-down under active and adaptive processing of higher-level knowledge and attention (e.g., Nusbaum and Schwab, 1986; Strait et al., 2010).

This conceptualization of speech perception as an active process has large repercussions for understanding the nature of hearing loss in older adults. Rabbitt (1991) has argued, as have others, that older adults, compared with younger adults, must employ additional perceptual and cognitive processing to offset sensory deficits in frequency and temporal resolution as well as in frequency range (Murphy et al., 2000; Pichora-Fuller and Souza, 2003; McCoy et al., 2005; Wingfield et al., 2005; Surprenant, 2007). Wingfield et al. (2005) have further argued that the use of this extra processing at the sensory level is costly and may affect the availability of cognitive resources that could be needed for other kinds of processing. While these researchers consider the cognitive consequences that may be encountered more generally given the demands on cognitive resources, such as the deficits found in the encoding of speech content in memory, there is less consideration of the way these demands may impact speech processing itself. If speech perception itself is mediated by active processes, which require cognitive resources, then the increasing demands on additional cognitive and perceptual processing for older adults becomes more problematic. The competition for cognitive resources may shortchange aspects of speech perception. Additionally, the difference between a passive system that simply involves the transduction, filtering, and simple pattern recognition (computing a distance between stored representations and input patterns and selecting the closest fit) and an active system that uses context dependent pattern recognition and signal-contingent adaptive processing has implications for the nature of augmentative hearing aids and programs of therapy for remediating aspects of hearing loss. It is well known that simple amplification systems are not sufficient remediation for hearing loss because they amplify noise as well as signal. Understanding how active processing operates and interacts with signal properties and cognitive processing might lead to changes in the way hearing aids operate, perhaps through cueing changes in attention, or by modifying the signal structure to affect the population coding of frequency information or attentional segregation of relevant signals. Training to use such hearing aids might be more effective by simple feedback or by systematically changing the level and nature of environmental sound challenges presented to listeners.

Furthermore, understanding speech perception as an active process has implications for explaining some of the findings of the interaction of hearing loss with cognitive processes (e.g., Wingfield et al., 2005). One explanation of the demands on cognitive mechanisms through hearing loss is a compensatory model as noted above (e.g., Rabbitt, 1991). This suggests that when sensory information is reduced, cognitive processes operate inferentially to supplement or replace the missing information. In many respects this is a kind of postperceptual explanation that might be like a response bias. It suggests that mechanisms outside of normal speech perception can be called on when sensory information is degraded. However an alternative view of the same situation is that it reflects the normal operation of speech recognition processing rather than an extra postperceptual inference system. Hearing loss may specifically exacerbate the fundamental problem of lack of invariance in acoustic-phonetic relationships.

The fundamental problem faced by all theories of speech perception derives from the lack of invariance in the relationship between the acoustic patterns of speech and the linguistic interpretation of those patterns. Although the many-to-many mapping between acoustic patterns of speech and perceptual interpretations is a longstanding well-known problem (e.g., Liberman et al., 1967), the core computational problem only truly emerges when a particular pattern has many different interpretations or can be classified in many different ways. It is widely established that individuals are adept in understanding the constituents of a given category, for traditional categories (Rosch et al., 1976) or *ad hoc* categories developed in response to the demands of a situation (Barsalou, 1983). In this sense, a many-to-one mapping does not pose a substantial computational challenge. As Nusbaum and Magnuson (1997) argue, a many-to-one mapping can be understood with a simple class of deterministic computational mechanisms. In essence, a deterministic system establishes one-to-one mappings between inputs and outputs and thus can be computed by passive mechanisms such as feature detectors. It is important to note that a many-to-one mapping (e.g., rising formant transitions signaling a labial stop and diffuse consonant release spectrum signaling a labial stop) can be instantiated as a collection of one-to-one mappings.

However, when a particular sensory pattern must be classified as a particular linguistic category and there are multiple possible interpretations, this constitutes a computational problem for recognition. In this case (e.g., a formant pattern that could signal either the vowel in BIT or BET) there is ambiguity about the interpretation of the input without additional information. One solution is that additional context or information could eliminate some alternative interpretations as in talker normalization (Nusbaum and Magnuson, 1997). But this leaves the problem of determining the nature of the constraining information and processing it, which is contingent on the ambiguity itself. This suggests that there is no automatic or passive means of identifying and using the constraining information. Thus an active mechanism, which tests hypotheses about interpretations and tentatively identifies sources of constraining information (Nusbaum and Schwab, 1986), may be needed.

Given that there are multiple alternative interpretations for a particular segment of speech signal, the nature of the information needed to constrain the selection depends on the source of variability that produced the one-to-many non-determinism. Variations in speaking rate, or talker, or linguistic context or other signal modifications are all potential sources of variability that are regularly encountered by listeners. Whether the system uses articulatory or linguistic information as a constraint, the perceptual system needs to flexibly use context as a guide in determining the relevant properties needed for recognition (Nusbaum and Schwab, 1986). The process of eliminating or weighing potential interpretations could well involve demands on working memory. Additionally, there may be changes in attention, towards more diagnostic patterns of information. Further, the system may be required to adapt to new sources of lawful variability in order to understand the context (cf. Elman and McClelland, 1986).

Generally speaking, these same kinds of mechanisms could be implicated in higher levels of linguistic processing in spoken language comprehension, although the neural implementation of such mechanisms might well differ. A many-to-many mapping problem extends to all levels of linguistic analysis in language comprehension and can be observed between patterns at the syllabic, lexical, prosodic and sentential level in speech and the interpretations of those patterns as linguistic messages. This is due to the fact that across linguistic contexts, speaker differences (idiolect, dialect, etc.) and other contextual variations, there are no patterns (acoustic, phonetic, syllabic, prosodic, lexical etc.) in speech that have an invariant relationship to the interpretation of those patterns. For this reason, it could be beneficial to consider how these phenomena of acoustic perception, phonetic perception, syllabic perception, prosodic perception, lexical perception, etc., are related computationally to one another and understand the computational similarities among the mechanisms that may subserve them (Marr, 1982). Given that such a mechanism needs to flexibly respond to changes in context (and different kinds of context—word or sentence or talker or speaking rate) and constrain linguistic interpretations in context, suggests that the mechanism for speech understanding needs to be plastic. In other words, speech recognition should inherently demonstrate learning.

## Learning mechanisms in speech

While on its face this seems uncontroversial, theories of speech perception have not traditionally incorporated learning although some have evolved over time to do so (e.g., Shortlist-B, Hebb-Trace). Indeed, there remains some disagreement about the plasticity of speech processing in adults. One issue is how the long-term memory structures that guide speech processing are modified to allow for this plasticity while at the same time maintaining and protecting previously learned information from being expunged. This is especially important as often newly acquired information may represent irrelevant information to the system in a long-term sense (Carpenter and Grossberg, 1988; Born and Wilhelm, 2012).

To overcome this problem, researchers have proposed various mechanistic accounts, and while there is no consensus amongst them, a hallmark characteristic of these accounts is that learning occurs in two stages. In the first stage, the memory system is able to use fast learning temporary storage to achieve adaptability, and in a subsequent stage, during an offline period such as sleep, this information is consolidated into long-term memory structures if the information is found to be germane (Marr, 1971; McClelland et al., 1995; Ashby et al., 2007). While this is a general cognitive approach to the formation of categories for recognition, this kind of mechanism does not figure into general thinking about speech recognition theories. The focus of these theories is less on the formation of category representations and the need for plasticity *during* recognition, than it is on the stability and structure of the categories (e.g., phonemes) to be recognized. Theories of speech perception often avoid the plasticity-stability trade off problem by proposing that the basic categories of speech are established early in life, tuned by exposure, and subsequently only operate as a passive detection system (e.g., Abbs and Sussman, 1971; Fodor, 1983; McClelland and Elman, 1986; although see Mirman et al., 2006b). According to these kinds of theories, early exposure to a system of speech input has important effects on speech processing.

Given the importance of early exposure for establishing the phonological system, there is no controversy regarding the significance of linguistic experience in shaping an individual’s ability to discriminate and identify speech sounds (Lisker and Abramson, 1964; Strange and Jenkins, 1978; Werker and Tees, 1984; Werker and Polka, 1993). An often-used example of this is found in how infants’ perceptual abilities change *via* exposure to their native language. At birth, infants are able to discriminate a wide range of speech sounds whether present or not in their native language (Werker and Tees, 1984). However, as a result of early linguistic exposure and experience, infants gain sensitivity to phonetic contrasts to which they are exposed and eventually lose sensitivity for phonetic contrasts that are not experienced (Werker and Tees, 1983). Additionally, older children continue to show developmental changes in perceptual sensitivity to acoustic-phonetic patterns (e.g., Nittrouer and Miller, 1997; Nittrouer and Lowenstein, 2007) suggesting that learning a phonology is not simply a matter of acquiring a simple set of mappings between the acoustic patterns of speech and the sound categories of language. Further, this perceptual learning does not end with childhood as it is quite clear that even adult listeners are capable of learning new phonetic distinctions not present in their native language (Werker and Logan, 1985; Pisoni et al., 1994; Francis and Nusbaum, 2002; Lim and Holt, 2011).

A large body of research has now established that adult listeners can learn a variety of new phonetic contrasts from outside their native language. Adults are able to learn to split a single native phonological category into two functional categories, such as Thai pre-voicing when learned by native English speakers (Pisoni et al., 1982) as well as to learn completely novel categories such as Zulu clicks for English speakers (Best et al., 1988). Moreover, adults possess the ability to completely change the way they attend to cues, for example Japanese speakers are able to learn the English /r/-/l/ distinction, a contrast not present in their native language (e.g., Logan et al., 1991; Yamada and Tohkura, 1992; Lively et al., 1993). While learning is limited, Francis and Nusbaum (2002) demonstrated that given appropriate feedback, listeners can learn to direct perceptual attention to acoustic cues that were not previously used to form phonetic distinctions in their native language. In their study, learning new categories was manifest as a change in the structure of the acoustic-phonetic space wherein individuals shifted from the use of one perceptual dimension (e.g., voicing) to a complex of two perceptual dimensions, enabling native English speakers to correctly perceive Korean stops after training. How can we describe this change? What is the mechanism by which this change in perceptual processing occurs?

From one perspective this change in perceptual processing can be described as a shift in attention (Nusbaum and Schwab, 1986). Auditory receptive fields may be tuned (e.g., Cruikshank and Weinberger, 1996; Weinberger, 1998; Wehr and Zador, 2003; Znamenskiy and Zador, 2013) or reshaped as a function of appropriate feedback (cf. Moran and Desimone, 1985) or context (Asari and Zador, 2009). This is consistent with theories of category learning (e.g., Schyns et al., 1998) in which category structures are related to corresponding sensory patterns (Francis et al., 2007, 2008). From another perspective this adaptation process could be described as the same kind of cue weighting observed in the development of phonetic categories (e.g., Nittrouer and Miller, 1997; Nittrouer and Lowenstein, 2007). Yamada and Tohkura (1992) describe native Japanese listeners as typically directing attention to acoustic properties of /r/-/l/ stimuli that are not the dimensions used by English speakers, and as such are not able to discriminate between these categories. This misdirection of attention occurs because these patterns are not differentiated functionally in Japanese as they are in English. For this reason, Japanese and English listeners distribute attention in the acoustic pattern space for /r/ and /l/ differently as determined by the phonological function of this space in their respective languages. Perceptual learning of these categories by Japanese listeners suggests a shift of attention to the English phonetically relevant cues.

This idea of shifting attention among possible cues to categories is part and parcel of a number of theories of categorization that are not at all specific to speech perception (e.g., Gibson, 1969; Nosofsky, 1986; Goldstone, 1998; Goldstone and Kersten, 2003) but have been incorporated into some theories of speech perception (e.g., Jusczyk, 1993). Recently, McMurray and Jongman (2011) proposed the C-Cure model of phoneme classification in which the relative importance of cues varies with context, although the model does not specify a mechanism by which such plasticity is implemented neurally.

One issue to consider in examining the paradigm of training non-native phonetic contrasts is that adult listeners bring an intact and complete native phonological system to bear on any new phonetic category-learning problem. This pre-existing phonological knowledge about the sound structure of a native language operates as a critical mass of an acoustic-phonetic system with which a new category likely does not mesh (Nusbaum and Lee, 1992). New contrasts can re-parse the acoustic cue space into categories that are at odds with the native system, can be based on cues that are entirely outside the system (e.g., clicks), or can completely remap native acoustic properties into new categories (see Best et al., 2001). In all these cases however listeners need to not only learn the pattern information that corresponds to these categories, but additionally learn the categories themselves. In most studies participants do not actually learn a completely new phonological system that exhibits an internal structure capable of supporting the acquisition of new categories, but instead learn isolated contrasts that are not part of their native system. Thus, learning non-native phonological contrasts requires individuals to learn both new category structures, as well as how to direct attention to the acoustic cues that define those categories without colliding with extant categories.

How do listeners accommodate the signal changes encountered on a daily basis in listening to speech? Echo and reverberation can distort speech. Talkers speak while eating. Accents can change the acoustic to percept mappings based on the articulatory phonetics of a native language. While some of the distortions in signals can probably be handled by some simple filtering in the auditory system, more complex signal changes that are systematic cannot be handled in this way. The use of filtering as a solution for speech signal distortion assumes a model of speech perception whereby a set of acoustic-phonetic representations (whether talker-specific or not) are obscured by some distortion and that some simple acoustic transform (like amplification or time-dilation) is used to restore the signal.

An alternative to this view was proposed by Elman and McClelland (1986). They suggested that the listener can use systematicity in distortions of acoustic patterns as information about the sources of variability that affected the signal in the conditions under which the speech was produced. This idea, that systematic variability in acoustic patterns of phonetic categories provides information about the intended phonetic message, suggests that even without learning new phonetic categories or contrasts, learning the sources and structure of acoustic-phonetic variability may be a fundamental aspect of speech perception. Nygaard et al. (1994) and Nygaard and Pisoni (1998) demonstrated that listeners learning the speech of talkers using the same phonetic categories as the listeners show significant improvements in speech recognition. Additionally, Dorman et al. (1977) elegantly demonstrated that different talkers speaking the same language can use different acoustic cues to make the same phonetic contrasts. In these situations, in order to recognize speech, listeners must learn to direct attention to the specific cues for a particular talker in order to ameliorate speech perception. In essence, this suggests that learning may be an intrinsic part of speech perception rather than something added on. Phonetic categories must remain plastic even in adults in order to flexibly respond to the changing demands of the lack of invariance problem across talkers and contexts of speaking.

One way of investigating those aspects of learning that are specific to directing attention to appropriate and meaningful acoustic cues without additionally having individuals learn new phonetic categories or a new phonological system, is to examine how listeners adapt to synthetic speech that uses their own native phonological categories. Synthetic speech generated by rule is “defective” in relation to natural speech in that it oversimplifies the acoustic pattern structure (e.g., fewer cues, less cue covariation) and some cues may actually be misleading (Nusbaum and Pisoni, 1985). Learning synthetic speech requires listeners to learn how acoustic information, produced by a particular talker, is used to define the speech categories the listener already possesses. In order to do this, listeners need to make use of degraded, sparse and often misleading acoustic information, which contributes to the poor intelligibility of synthesized speech. Given that such cues are not available to awareness, and that most of such learning is presumed to occur early in life, it seems difficult to understand that adult listeners could even do this. In fact, it is this ability to rapidly learn synthetic speech that lead Nusbaum and Schwab (1986) to conclude that speech must be guided by active control processes.

## Generalization learning

In a study reported by Schwab et al. (1985), listeners were trained on synthetic speech for 8 days with feedback and tested before and after training. Before training, recognition was about 20% correct, but improved after training to about 70% correct. More impressively this learning occurred even though listeners were never trained or tested on the same words twice, meaning that individuals had not just explicitly learned what they were trained on, but instead gained generalized knowledge about the synthetic speech. Additionally, Schwab et al. (1985) demonstrated that listeners are able to substantially retain this generalized knowledge without any additionally exposure to the synthesizer, as listeners showed similar performance 6 months later. This suggests that even without hearing the same words over and over again, listeners were able to change the way they used acoustic cues at a sublexical level. In turn, listeners used this sublexical information to drive recognition of these cues in completely novel lexical contexts. This is far different from simply memorizing the specific and complete acoustic patterns of particular words, but instead could reflect a kind of procedural knowledge of how to direct attention to the speech of the synthetic talker.

This initial study demonstrated clear generalization beyond the specific patterns heard during training. However on its own it gives little insight into the way such generalization emerges. In a subsequent study, Greenspan et al. (1988) expanded on this and examined the ability of adult listeners to generalize from various training regimes asking the question of how acoustic-phonetic variability affects generalization of speech learning. Listeners were either given training on repeated words or novel words, and when listeners memorize specific acoustic patterns of spoken words, there is very good recognition performance for those words. However this does not afford the same level of perceptual generalization that is produced by highly variable training experiences. This is akin to the benefits of training variability seen in motor learning in which generalization of a motor behavior is desired (e.g., Lametti and Ostry, 2010; Mattar and Ostry, 2010; Coelho et al., 2012). Given that training set variability modulates the type of learning, adult perceptual learning of spoken words cannot be seen as simply a rote process. Moreover, even from a small amount of repeated and focused rote training there is some reliable generalization indicating that listeners can use even restricted variability in learning to go beyond the training examples (Greenspan et al., 1988). Listeners may infer this generalized information from the training stimuli, or they might develop a more abstract representation of sound patterns based on variability in experience and apply this knowledge to novel speech patterns in novel contexts.

Synthetic speech, produced by rule, as learned in those studies, represents a complete model of speech production from orthographic-to-phonetic-to-acoustic generation. The speech that is produced is recognizable but it is artificial. Thus learning of this kind of speech is tantamount to learning a strange idiolect of speech that contains acoustic-phonetic errors, missing acoustic cues and does not possess correct cue covariation. However if listeners learn this speech by gleaning the new acoustic-phonetic properties for this kind of talker, it makes sense that listeners should be able to learn other kinds of speech as well. This is particularly true if learning is accomplished by changing the way listeners attend to the acoustic properties of speech by focusing on the acoustic properties that are most phonetically diagnostic. And indeed, beyond being able to learn synthesized speech in this fashion, adults have been shown to quickly adapt to a variety of other forms of distorted speech where the distortions initially cause a reduction in intelligibility, such as simulated cochlear implant speech (Shannon et al., 1995), spectrally shifted speech (Rosen et al., 1999) as well as foreign-accented speech (Weil, 2001; Clarke and Garrett, 2004; Bradlow and Bent, 2008; Sidaras et al., 2009). In these studies, listeners learn speech that has been produced naturally with coarticulation and the full range of acoustic-phonetic structure, however, the speech signal deviates from listener expectations due to a transform of some kind, either through signal processing or through phonological changes in speaking. Different signal transforms may distort or mask certain cues and phonological changes may change cue complex structure. These distortions are unlike synthetic speech however, as these transforms tend to be uniform across the phonological inventory. This would provide listeners with a kind of lawful variability (as described by Elman and McClelland, 1986) that can be exploited as an aid to recognition. Given that in all these speech distortions listeners showed a robust ability to apply what they learned during training to novel words and contexts, learning does not appear to be simply understanding what specific acoustic cues mean, but rather understanding what acoustic cues are most relevant for a given source and how to attend to them (Nusbaum and Lee, 1992; Nygaard et al., 1994; Francis and Nusbaum, 2002).

How do individuals come to learn what acoustic cues are most diagnostic for a given source? One possibility is that acoustic cues are mapped to their perceptual counterparts in an unguided fashion, that is, without regard for the systematicity of native acoustic-phonetic experience. Conversely, individuals may rely on their native phonological system to guide the learning process. In order to examine if perceptual learning is influenced by an individual’s native phonological experience, Davis et al. (2005) examined if perceptual learning was more robust when individuals were trained on words versus non-words. Their rationale was that if training on words led to better perceptual learning than non-words, then one could conclude that the acoustic to phonetic remapping process is guided or structured by information at the lexical level. Indeed, Davis et al. (2005) showed that training was more effective when the stimuli consisted of words than non-words, indicating that information at the lexical level allows individuals to use their knowledge about how sounds are related in their native phonological system to guide the perceptual learning process. The idea that perceptual learning in speech is driven to some extent by lexical knowledge is consistent with both autonomous (e.g., Shortlist: Norris, 1994; Merge: Norris et al., 2000; Shortlist B: Norris and McQueen, 2008) and interactive (e.g., TRACE: McClelland and Elman, 1986; Hebb-Trace: Mirman et al., 2006a) models of speech perception (although whether learning can successfully operate in these models is a different question altogether). A subsequent study by Dahan and Mead (2010) examined the structure of the learning process further by asking how more localized or recent experience, such as the specific contrasts present during training, may organize and determine subsequent learning. To do this, Dahan and Mead (2010) systematically controlled the relationship between training and test stimuli as individuals learned to understand noise vocoded speech. Their logic was that if localized or recent experience organizes learning, then the phonemic contrasts present during training may provide such a structure, such that phonemes will be better recognized at test if they had been heard in a similar syllable position or vocalic context during training than if they had been heard in a different context. Their results showed that individuals’ learning was directly related to the local phonetic context of training, as consonants were recognized better if they had been heard in a similar syllable position or vocalic context during training than if they had been heard in a dissimilar context.

This is unsurprising as the acoustic realization of a given consonant can be dramatically different depending on the position of a consonant within a syllable (Sproat and Fujimura, 1993; Browman and Goldstein, 1995). Further, there are coarticulation effects such that the acoustic characteristics of a consonant are heavily modified by the phonetic context in which it occurs (Liberman et al., 1954; Warren and Marslen-Wilson, 1987; Whalen, 1991). In this sense, the acoustic properties of speech are not dissociable beads on a string and as such, the linguistic context of a phoneme is very much apart of the acoustic definition of a phoneme. While experience during training does appear to be the major factor underlying learning, individuals also show transfer of learning to phonemes that were not presented during training provided that were perceptually similar to the phonemes that were present. This is consistent with a substantial body of speech research using perceptual contrast procedures that showed that there are representations for speech sounds both at the level of the allophonic or acoustic-phonetic specification as well as at a more abstract phonological level (e.g., Sawusch and Jusczyk, 1981; Sawusch and Nusbaum, 1983; Hasson et al., 2007). Taken together both the Dahan and Mead (2010) and the Davis et al. (2005) studies provide clear evidence that previous experience, such as the knowledge of one’s native phonological system, as well as more localized experience relating to the occurrence of specific contrasts in a training set help to guide the perceptual learning process.

What is the nature of the mechanism underlying the perceptual learning process that leads to better recognition after training? To examine if training shifts attention to phonetically meaningful cues and away from misleading cues, Francis et al. (2000), trained listeners on CV syllables containing /b/, /d/, and or /g/ cued by a chimeric acoustic structure containing either consistent or conflicting properties. The CV syllables were constructed such that the place of articulation was specified by the spectrum of the burst (Blumstein and Stevens, 1980) as well as by the formant transitions from the consonant to the vowel (e.g., Liberman et al., 1967). However, for some chimeric CVs, the spectrum of the burst indicated a different place of articulation than the transition cue. Previously Walley and Carrell (1983) had demonstrated that listeners tend to identify place of articulation based on transition information rather than the spectrum of the burst when these cues conflict. And of course listeners never consciously hear either of these as separate signals—they simply hear a consonant at a particular place of articulation. Given that listeners cannot identify the acoustic cues that define the place of articulation consciously and only experience the categorical identity of the consonant itself, it seems hard to understand how attention can be directed towards these cues.

Francis et al. (2000) trained listeners to recognize the chimeric speech in their experiment by providing feedback about the consonant identity that was either consistent with the burst cues or the transition cues depending on the training group. For the burst-trained group, when listeners heard a CV and identified it as a B, D, or G, they would receive feedback following identification. For a chimeric consonant cued with a labial burst and an alveolar transition pattern (combined), whether listeners identified the consonant as B (correct for the burst-trained group) or another place, after identification they would hear the CV again and see feedback printed identifying the consonant as B. In other words, burst-trained listeners would get feedback during training consistent with the spectrum of the burst whereas transition-trained listeners would get feedback consistent with the pattern of the transitions. The results showed that cue-based feedback shifted identification performance over training trials such that listeners were able to learn to use the specific cue (either transition based or spectral burst based) that was consistent with the feedback and generalized to novel stimuli. This kind of learning research (also Francis and Nusbaum, 2002; Francis et al., 2007) suggests shifting attention may serve to restructure perceptual space as a result of appropriate feedback.

Although the standard view of speech perception is one that does not explicitly incorporate learning mechanisms, this is in part because of a very static view of speech recognition whereby stimulus patterns are simply mapped onto phonological categories during recognition, and learning may occur, if it does, afterwards. These theories never directly solve the lack of invariance problem, given a fundamentally deterministic computational process in which input states (whether acoustic or articulatory) must correspond uniquely to perceptual states (phonological categories). An alternative is to consider speech perception is an active process in which alternative phonetic interpretations are activated, each corresponding to a particular input pattern from speech (Nusbaum and Schwab, 1986). These alternatives must then be reduced to the recognized form, possibly by testing these alternatives as hypotheses shifting attention among different aspects of context, knowledge, or cues to find the best constraints. This view suggests that there should be an increase in cognitive load on the listener until a shift of attention to more diagnostic information occurs when there is a one-to-many mapping, either due to speech rate variability (Francis and Nusbaum, 1996) or talker variability (Nusbaum and Morin, 1992). Variation in talker or speaking rate or distortion can change the way attention is directed at a particular source of speech, shifting attention towards the most diagnostic cues and away from the misleading cues. This suggests a direct link between attention and learning, with the load on working memory reflecting the uncertainty of recognition given a one-to-many mapping of acoustic cues to phonemes.

If a one-to-many mapping increases the load on working memory because of active alternative phonetic hypotheses, and learning shifts attention to more phonetically diagnostic cues, learning to perceive synthetic speech should reduce the load on working memory. In this sense, focusing attention on the diagnostic cues should reduce the number of phonetic hypotheses. Moreover, this should not simply be a result of improved intelligibility, as increasing speech intelligibility without training should not have the same effect. To investigate this, Francis and Nusbaum (2009) used a speeded spoken target monitoring procedure and manipulated memory load to see if the effect of such a manipulation would change as a function of learning synthetic speech. The logic of the study was that varying a working memory load explicitly should affect recognition speed if working memory plays a role in recognition. Before training, working memory should have a higher load than after training, suggesting that there should be an interaction between working memory load and the training in recognition time (cf. Navon, 1984). When the extrinsic working memory load (to the speech task) is high, there should be less working memory available for recognition but when the extrinsic load is low there should be more working memory available. This suggests that training should interact with working memory load by showing a larger improvement of recognition time in the low load case than in the high load case. Of course if speech is directly mapped from acoustic cues to phonetic categories, there is no reason to predict a working memory load effect and certainly no interaction with training. The results demonstrated however a clear interaction of working memory load and training as predicted by the use of working memory and attention (Francis and Nusbaum, 2009). These results support the view that training reorganizes perception, shifting attention to more informative cues allowing working memory to be used more efficiently and effectively. This has implications for older adults who suffer from hearing loss. If individuals recruit additional cognitive and perceptual resources to ameliorate sensory deficits, then they will lack the necessary resources to cope with situations where there is an increase in talker or speaking rate variability. In fact, Peelle and Wingfield (2005) report that while older adults can adapt to time-compressed speech, they are unable to transfer learning on one speech rate to a second speech rate.

## Mechanisms of Memory

Changes in the allocation of attention and the demands on working memory are likely related to substantial modifications of category structures in long term memory (Nosofsky, 1986; Ashby and Maddox, 2005). Effects of training on synthetic speech have been shown to be retained for 6 months suggesting that categorization structures in long-term memory that guide perception have been altered (Schwab et al., 1985). How are these category structures that guide perception (Schyns et al., 1998) modified? McClelland and Rumelhart (1985) and McClelland et al. (1995) have proposed a neural cognitive model that explains how individuals are able to adapt to new information in their environment. According to their model, specific memory traces are initially encoded during learning via a fast-learning hippocampal based memory system. Then, *via* a process of repeated reactivation or rehearsal, memory traces are strengthened and ultimately represented solely in the neocortical memory system. One of the main benefits of McClelland’s model is that it explains how previously learned information is protected against newly acquired information that may potentially be irrelevant for long-term use. In their model, the hippocampal memory system acts as temporary storage where fast-learning occurs, while the neocortical memory system, which houses the long-term memory category that guide perception, are modified later, presumably offline when there are no encoding demands on the system. This allows the representational system to remain adaptive without the loss of representational stability as only memory traces that are significant to the system will be strengthened and rehearsed. This kind of two-stage model of memory is consistent with a large body of memory data, although the role of the hippocampus outlined in this model is somewhat different than other theories of memory (e.g., Eichenbaum et al., 1992; Wood et al., 1999, 2000).

Ashby et al. (2007) have also posited a two-stage model for category learning, but implementing the basis for the two stages, as well as their function in category formation, very differently. They suggest that the basal ganglia and the thalamus, rather than the hippocampus, together mediate the development of more permanent neorcortical memory structures. In their model, the striatum, globus pallidus, and thalamus comprise the fast learning temporary memory system. This subcortical circuit is has greater adaptability due to the dopamine-mediated learning that can occur in the basal ganglia, while representations in the neocortical circuit are much more slow to change as they rely solely on Hebbian learning to be amended.

McClelland’s neural model relies on the hippocampal memory system as a substrate to support the development of the long-term memory structures in neocortex. Thus hippocampal memories are comprised of recent specific experiences or rote memory traces that are encoded during training. In this sense, the hippocampal memory circuit supports the longer-term reorganization or consolidation of declarative memories. In contrast, in the basal ganglia based model of learning put forth by Ashby a striatum to thalamus circuit provides the foundation for the development of consolidation in cortical circuits. This is seen as a progression from a slow based hypothesis-testing system to a faster processing, implicit memory system. Therefore the striatum to thalamus circuit mediates the reorganization or consolidation of procedural memories. To show evidence for this, Ashby et al. (2007) use information-integration categorization tasks, where the rules that govern the categories that are to be learned are not easily verbalized. In these tasks, the learner is required to integrate information from two or more dimensions at some pre-decisional stage. The logic is that information-integration tasks use the dopamine-mediated reward signals afforded by the basal ganglia. In contrast, in rule-based categorization tasks the categories to be learned are explicitly verbally defined, and thus rely on conscious hypothesis generation and testing. As such, this explicit category learning is thought (Ashby et al., 2007) to be mediated by the anterior cingulate and the prefrontal cortex. For this reason, demands on working memory and executive attention are hypothesized to affect only the learning of explicit based categories and not implicit procedural categories, as working memory and executive attention are processes that are largely governed by the prefrontal cortex (Kane and Engle, 2000).

The differences between McClelland and Ashby’s models appear to be related in part to the distinction between declarative versus procedural learning. While it is certainly reasonable to divide memory in this way, it is unarguable that both types of memories involve encoding and consolidation. While it may be the case that the declarative and procedural memories operate through different systems, this seems unlikely given that there are data suggesting the role of the hippocampus in procedural learning (Chun and Phelps, 1999) even when this is not a verbalizable and an explicit rule-based learning process. Elements of the theoretic assumptions of both models seem open to criticism in one way or another. But both models make explicit a process by which rapidly learned, short-term memories can be consolidated into more stable forms. Therefore it is important to consider such models in trying to understand the process by which stable memories are formed as the foundation of phonological knowledge in speech perception.

As noted previously, speech appears to have separate representations for the specific acoustic patterns of speech as well as more abstract phonological categories (e.g., Sawusch and Jusczyk, 1981; Sawusch and Nusbaum, 1983; Hasson et al., 2007). Learning appears to occur at both levels as well (Greenspan et al., 1988) suggesting the importance of memory theory differentiating both short-term and long-term representations as well as stimulus specific traces and more abstract representations. It is widely accepted that any experience may be represented across various levels of abstraction. For example, while only specific memory traces are encoded for many connectionist models (e.g., McClelland and Rumelhart’s 1985 model), various levels of abstraction can be achieved in the retrieval process depending on the goals of the task. This is in fact the foundation of Goldinger’s (1998) echoic trace model based on Hintzman’s (1984) MINERVA2 model. Specific auditory representations of the acoustic pattern of a spoken word are encoded into memory and abstractions are derived during the retrieval process using working memory.

In contrast to these trace-abstraction models is another possibility wherein stimulus-specific and abstracted information are both stored in memory. For example an acoustic pattern description of speech as well as a phonological category description are represented separately in memory in the TRACE model (McClelland and Elman, 1986; Mirman et al., 2006a). In this respect then, the acoustic patterns of speech—as particular representations of a specific perceptual experience—are very much like the echoic traces of Goldinger’s model. However where Goldinger argued against forming and storing abstract representations, others have suggested that such abstractions may in fact be formed and stored in the lexicon (see Luce et al., 2003; Ju and Luce, 2006). Indeed, Hasson et al. (2007) demonstrated repetition suppression effects specific to the abstract phonological representation of speech sounds given that the effect held between an illusory syllable /ta/ and a physical syllable /ta/ based on a network spanning sensory and motor cortex. Such abstractions are unlikely to simply be an assemblage of prior sensory traces given that the brain areas involved are not the same as those typically activated in recognizing those traces. In this way, memory can be theoretically distinguished into rote representational structures that consist of specific experienced items or more generalized structures that consist of abstracted information. Rote memories are advantageous for precise recall of already experienced stimuli where as generalized memory would favor performance for a larger span of stimuli in a novel context.

This distinction between rote and generalized representations cuts across the distinction between procedural and declarative memory. Both declarative and procedural memories may be encoded as either rote or generalized memory representational structures. For example, an individual may be trained to press a specific sequence of keys on a keyboard. This would lead to the development of a rote representational memory structure, allowing the individual to improve his or her performance on that specific sequence. Alternatively, the individual may be trained to press several sequences of keys on a keyboard. This difference in training would lead to the development of a more generalized memory structure, resulting in better performance both experienced and novel key sequences. Similarly declarative memories may be encoded as either rote or generalized structures as a given declarative memory structures may consist of either the specific experienced instances of a particular stimulus, as in a typical episodic memory experiment, or the “gist” of the experienced instances as in the formation of semantic memories or possibly illusory memories based on associations (see Gallo, 2006).

The argument about the distinction between rote and generalized or abstracted memory representations becomes important when considering the way in which memories become stabilized through consolidation. In particular, for perceptual learning of speech, two aspects are critical. First, given the generativity of language and the context-sensitive nature of acoustic-phonetics, listeners are not going to hear the same utterances again and again and further, the acoustic pattern variation in repeated utterances, even if they occurred, would be immense due to changes in linguistic context, speaking rate, and talkers. As such, this makes the use of rote-memorization of acoustic patterns untenable as a speech recognition system. Listeners either have to be able to generalize in real time from prior auditory experiences (as suggested by Goldinger, 1998) or there must be more abstract representations that go beyond the specific sensory patterns of any particular utterance (as suggested by Hasson et al., 2007). This is unlikely due to the second consideration, which is that any generalizations in speech perception must be made quickly and remain stable to be useful. As demonstrated by Greenspan et al. (1988) even learning a small number of spoken words from a particular speech synthesizer will produce some generalization to novel utterances, although increasing the variability in experiences will increase the amount of generalization.

The separation between rote and generalization learning is further demonstrated by the effects of sleep consolidation on the stability of memories. In the original synthetic speech learning study by Schwab et al. (1985), listeners demonstrated significant learning in spite of never hearing the same words twice. Moreover this generalization learning lasted for roughly 6 months without subsequent training. This demonstrates that high variability in training examples with appropriate feedback can produce large improvements in generalized performance that can remain robust and stable for a long time. Fenn et al. (2003) demonstrated that this stability is a consequence of sleep consolidation of learning. In addition, when some forgetting takes place over the course of a day following learning, sleep restores the forgotten memories. It appears that this may well be due to sleep separately consolidating both the initial learning as well as any interference that occurs following learning (Brawn et al., 2013). Furthermore, Fenn and Hambrick (2012) have demonstrated that the effectiveness of sleep consolidation is related to individual differences in working memory such that higher levels of working memory performance are related to better consolidation. This links the effectiveness of sleep consolidation to a mechanism closely tied to active processing in speech perception. Most recently though, Fenn et al. (2013) found that sleep operates differently for rote and generalized learning.

These findings have several implications for therapy with listeners with hearing loss. First, training and testing should be separated by a period of sleep in order to measure the amount of learning that is stable. Second, although variability in training experiences seems to produce slower rates of learning, it produces greater generalization learning. Third, measurements of working memory can give a rough guide to the relative effectiveness of sleep consolidation thereby indicating how at risk learning may be to interference and suggesting that training may need to be more prolonged for people with lower working memory capacity.

## Conclusion

Theories of speech perception have often conceptualized the earliest stages of auditory processing of speech to be independent of higher level linguistic and cognitive processing. In many respects this kind of approach (e.g., in Shortlist B) treats the phonetic processing of auditory inputs as a passive system in which acoustic patterns are directly mapped onto phonetic features or categories, albeit with some distribution of performance. Such theories treat the distributions of input phonetic properties as relatively immutable. However, our argument is that even early auditory processes are subject to descending attentional control and active processing. Just as echolocation in the bat is explained by a cortofugal system in which cortical and subcortical structures are viewed as processing cotemporaneously and interactively (Suga, 2008), the idea that descending projects from cortex to thalamus and to the cochlea provide a neural substrate for cortical tuning of auditory inputs. Descending projections from the lateral olivary complex to the inner hair cells and from the medial olivary complex to the outer hair cells provide a potential basis for changing auditory encoding in real time as a result of shifts of attention. This kind of mechanism could support the kinds of effects seen in increased auditory brainstem response fidelity to acoustic input following training (Strait et al., 2010).

Understanding speech perception as an active process suggests that learning or plasticity is not simply a higher-level process grafted on top of word recognition. Rather the kinds of mechanisms involved in shifting attention to relevant acoustic cues for phoneme perception (e.g., Francis et al., 2000, 2007) are needed for tuning speech perception to the specific vocal characteristics of a new speaker or to cope with distortion of speech or noise in the environment. Given that such plasticity is linked to attention and working memory, we argue that speech perception is inherently a cognitive process, even in terms of the involvement of sensory encoding. This has implications for remediation of hearing loss either with augmentative aids or therapy. First, understanding the cognitive abilities (e.g., working memory capacity, attention control etc.) may provide guidance on how to design a training program by providing different kinds of sensory cues that are correlated or reducing the cognitive demands of training. Second, increasing sensory variability within the limits of individual tolerance should be part of a therapeutic program. Third, understanding the sleep practice of participants using sleep logs, record of drug and alcohol consumption, and exercise are important to the consolidation of learning. If speech perception is continuously plastic but there are limitations based on prior experiences and cognitive capacities, this shapes the basic nature of remediation of hearing loss in a number of different ways.

Finally, we would note that there is a dissociation among the three classes of models that are relevant to understanding speech perception as an active process. Although cognitive models of spoken word processing (e.g., Cohort, TRACE, and Shortlist) have been developed to include some plasticity and to account for different patterns of the influence of lexical knowledge, even the most recent versions (e.g., Distributed Cohort, Hebb-TRACE, and Shortlist B) do not specifically account for active processing of auditory input. It is true that some models have attempted to account for active processing below the level of phonemes (e.g., TRACE I: Elman and McClelland, 1986; McClelland et al., 2006), these models not been related or compared systematically to the kinds of models emerging from neuroscience research. For example, Friederici (2012) and Rauschecker and Scott (2009) and Hickok and Poeppel (2007) have all proposed neurally plausible models largely around the idea of dorsal and ventral processing streams. Although these models differ in details, in principle the model proposed by Friederici (2012) and Rauschecker and Scott (2009) have more extensive feedback mechanisms to support active processing of sensory input. These models are constructed in a neuroanatomical vernacular rather than the cognitive vernacular (even the Hebb-TRACE is still largely a cognitive model) of the others. But both sets of models are notable for two important omissions.

First, while the cognitive models mention learning and even model it, and the neural models refer to some aspects of learning, these models do not relate to the two-process learning models (e.g., complementary learning systems (CLS; McClelland et al., 1995; Ashby and Maddox, 2005; Ashby et al., 2007)). Although CLS focuses on episodic memory and Ashby et al. (2007) focus on category learning, two process models involving either hippocampus, basal ganglia, or cerebellum as a fast associator and cortico-cortical connections as a slower more robust learning system, have garnered substantial interest and research support. Yet learning in the models of speech recognition has yet to seriously address the neural bases of learning and memory except descriptively.

This points to a second important omission. All of the speech recognition models are cortical models. There is no serious consideration to the role of the thalamus, amygdala, hippocampus, cerebellum or other structures in these models. In taking a corticocentric view (see Parvizi, 2009), these models exhibit an unrealistic myopia about neural explanations of speech perception. Research by Kraus et al. (Wong et al., 2007; Song et al., 2008) demonstrates that there are measurable effects of training and experience on speech processing in the auditory brainstem. This is consistent with an active model of speech perception in which attention and experience shape the earliest levels of sensory encoding of speech. Although current data do not exist to support online changes in this kind of processing, this is exactly the kind of prediction an active model of speech perception would make but is entirely unexpected from any of the current models of speech perception.

## Author contributions

Shannon L. M. Heald prepared the first draft and Howard C. Nusbaum revised and both refined the manuscript to final form.

## Conflict of interest statement

The authors declare that the research was conducted in the absence of any commercial or financial relationships that could be construed as a potential conflict of interest.
